# Truncated DNM1 variant underlines developmental delay and epileptic encephalopathy

**DOI:** 10.3389/fped.2023.1266376

**Published:** 2023-10-09

**Authors:** Tayyaba Afsar, Xiaoyun Huang, Abid Ali Shah, Safdar Abbas, Shazia Bano, Arif Mahmood, Junjian Hu, Suhail Razak, Muhammad Umair

**Affiliations:** ^1^ Department of Community Health Sciences, College of Applied Medical Sciences, King Saud University, Riyadh, Saudi Arabia; ^2^ King Salman Center for Disability Research, Riyadh, Saudi Arabia; ^3^ Department of Neurology, SSL Central Hospital of Dongguan City, Affiliated Dongguan Shilong People’s Hospital of Southern Medical University, Dongguan, China; ^4^ Center for Medical Genetics and Hunan Key Laboratory of Medical Genetics, School of Life Sciences, Central South University, Changsha, China; ^5^ Department of Biological Sciences, Dartmouth College, Hanover, NH, United States; ^6^ Department of Optometry and Vision Sciences, University of Lahore, Lahore, Pakistan; ^7^ Department of Central Laboratory, SSL Central Hospital of Dongguan City, Affiliated Dongguan Shilong People’s Hospital of Southern Medical University, Dongguan, China; ^8^ Medical Genomics Research Department, King Abdullah International Medical Research Center (KAIMRC), King Saud Bin Abdulaziz University for Health Sciences, Ministry of National Guard Health Affairs (MNGH), Riyadh, Saudi Arabia

**Keywords:** *DNM1*, homozygous variant, non-sense variant, developmental and epileptic encephalopathies (DEEs), novel mutation

## Abstract

**Background:**

Developmental and epileptic encephalopathies (DEEs) signify a group of heterogeneous neurodevelopmental disorder associated with early-onset seizures accompanied by developmental delay, hypotonia, mild to severe intellectual disability, and developmental regression. Variants in the *DNM1* gene have been associated with autosomal dominant DEE type 31A and autosomal recessive DEE type 31B.

**Methods:**

In the current study, a consanguineous Pakistani family consisting of a proband (IV-2) was clinically evaluated and genetically analyzed manifesting in severe neurodevelopmental phenotypes. WES followed by Sanger sequencing was performed to identify the disease-causing variant. Furthermore, 3D protein modeling and dynamic simulation of wild-type and mutant proteins along with reverse transcriptase (RT)–based mRNA expression were checked using standard methods.

**Results:**

Data analysis of WES revealed a novel homozygous non-sense variant (c.1402G>T; p. Glu468*) in exon 11 of the *DNM1* gene that was predicted as pathogenic class I. Variants in the *DNM1* gene have been associated with DEE types 31A and B. Different bioinformatics prediction tools and American College of Medical Genetics guidelines were used to verify the identified variant. Sanger sequencing was used to validate the disease-causing variant. Our approach validated the pathogenesis of the variant as a cause of heterogeneous neurodevelopmental disorders. In addition, 3D protein modeling showed that the mutant protein would lose most of the amino acids and might not perform the proper function if the surveillance non-sense-mediated decay mechanism was skipped. Molecular dynamics analysis showed varied trajectories of wild-type and mutant DNM1 proteins in terms of root mean square deviation, root mean square fluctuation and radius of gyration. Similarly, RT-qPCR revealed a substantial reduction of the *DNM1* gene in the index patient.

**Conclusion:**

Our finding further confirms the association of homozygous, loss-of-function variants in *DNM1* associated with DEE type 31B. The study expands the genotypic and phenotypic spectrum of pathogenic *DNM1* variants related to *DNM1*-associated pathogenesis.

## Introduction

Developmental and epileptic encephalopathy (DEE) are a group of genetic conditions characterized by severe epilepsy affecting children, resulting in delayed development, neurological and non-neurological comorbidities, and, in rare cases, early mortality (1–3). More than 100 monogenic causes of epilepsies and neurodevelopmental abnormalities have been identified, and genetic factors have since been recognized as significant contributors to DEEs (4, 5).

The majority of genetics-based epilepsies and neurodevelopmental abnormalities are caused by variants in genes involved in synaptic transmission. Several genes involved in synaptic vesicle fission and fusion, such as *AP2M1* (MIM: 601024), *DNM1* (MIM: 602377), *STX1B* (MIM: 601485), *STXBP1* (MIM: 602926), *SNAP25* (MIM: 600322), and *VAMP2* (MIM: 185881), have been implicated in neurodevelopmental disorders (6–12). Among these, deleterious variants in the *DNM1* gene have been responsible for diverse neurodevelopmental phenotypes and cerebral dysfunctions such as early-onset epileptic encephalopathies. These conditions usually occur at an early period of life accompanying infantile spasms, developmental delay, and movement disorders (11). In general, disease-causing *DNM1* variants are responsible for around 2% of patients with infantile spasms or the Lennox–Gastaut syndrome (13).

Dynamin 1 (DNM1; NM_004408), localized to chromosome 9q34.11, encodes an 864 amino acid DNM1 protein, which is a GTPase-binding protein, and performs the function of synaptic vesicle fission for receptor-mediated endocytosis in the presynaptic plasma membrane (14). It consist of five domains: (a) an N-terminal G domain (GTPase domain); (b) a middle domain, involved in oligomerization; (c) a pleckstrin homology (PH) domain; (d) GTPase effector domain (GED); and (e) a proline-rich domain (PRD) (15).

Majority of the *de novo* deleterious variants occur in the only two GTPase and middle domains manifested in patients with epileptic encephalopathies (11, 16, 17). Only one deleterious variant c.1603A>G (p.Lys535Glu) has been observed in the PH domain in twin siblings positive for intellectual disability (ID) and autistic symptoms but no epileptic encephalopathy (18).

Neurological abnormalities are a highly heterogeneous group of rare genetic disorders that are primarily diagnosed using whole exome sequencing (WES) (19). Here, we describe a patient harboring a novel *de novo* non-sense pathogenic variant in the middle domain of the DNM1 protein, whose phenotypes included moderate ID, with mild generalized epilepsy with onset in adolescence.

## Materials and methods

### Ethics statement and study approval

**Figure 1:**
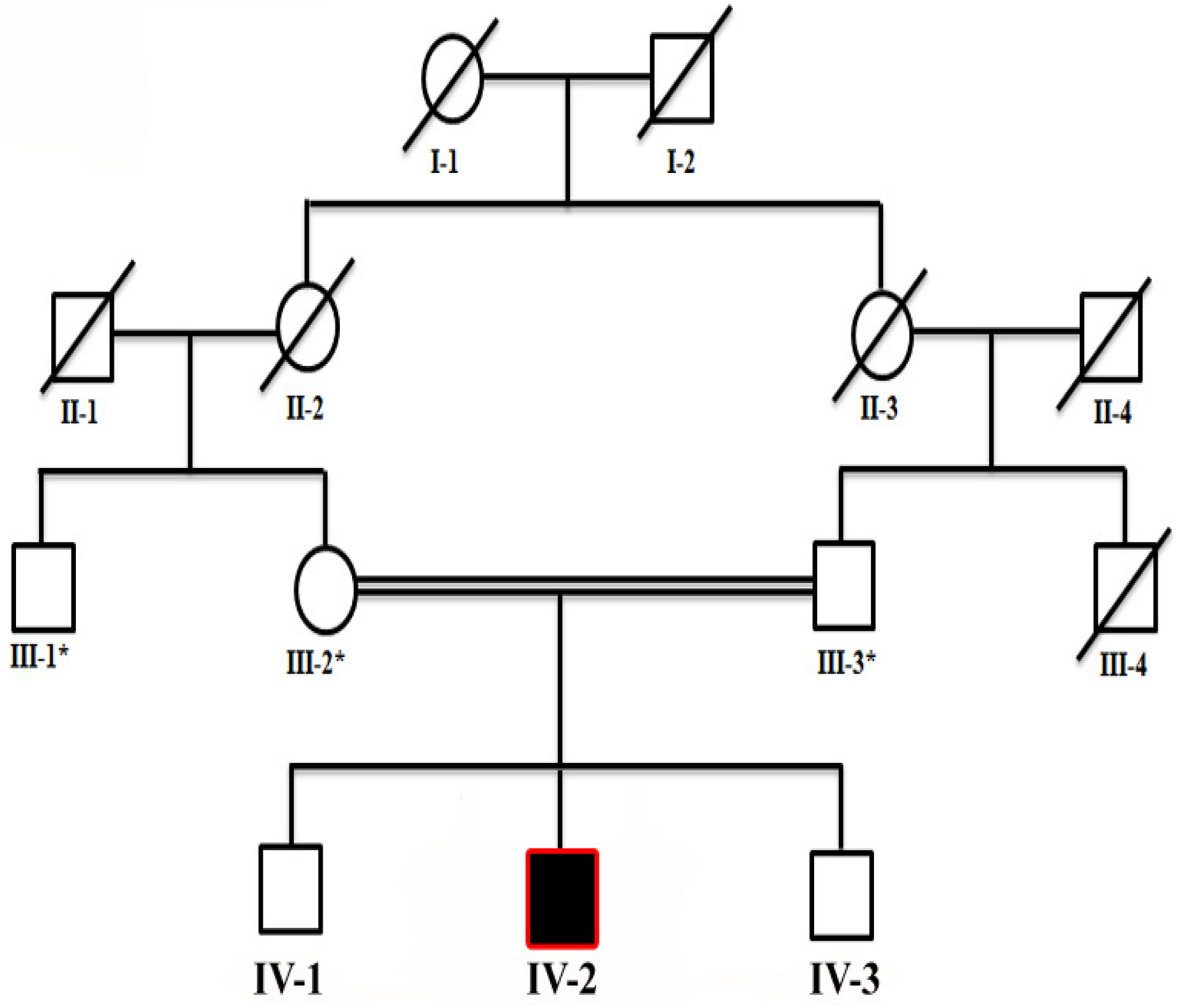
Four-generation pedigree of a family in the present study showing consanguinity (double lines) and autosomal recessive inheritance of the disease. The patient is highlighted with a black square, while an asterisk sign represents individuals subjected for WES. Slashes represent diseased individuals.

The study presented here describes a proband (IV-2) suffering from a rare neurodevelopmental disorder. The family of the proband showing neurodevelopmental phenotypes was recruited from a remote region of Khyber Pakhtunkhwa (KP), Pakistan. Before the study, the parents were interviewed and patient history was obtained (Figure 1). Informed consent in written form was obtained from the patients and other family members for the publication of the data, results, molecular findings, X-rays, and other related data in this article in compliance with the Helsinki Declaration. Blood samples were collected in Ethylenediaminetetraacetic acid (EDTA) tubes. DNA from blood samples was extracted and quantified using standard methods described previously (20).

### Molecular analysis

#### Chromosomal and cytogenetic analysis

Chromosomal and cytogenetic analysis was performed using standard methods as described previously (21).

#### Whole exome sequencing

WES was performed using DNA from the affected member (II-I). WES and variants filtering steps were performed as described earlier (22, 23), using the Illumina Hi-seq-2500 platform following the manufacturer instructions. After the standard reaction, The data was obtained and evaluated using the bioinformatics procedure into the variant call format (VCF). The VCF was then uploaded into the professional data analysis software (https://basespace.illumina.com/) to filter out the variants. Standard screening principles were used to search for different functional variants.

#### Determination of variant pathogenicity

The identified variant was first searched in general population databases such as gnomAD (24) and an in-house database to rule out the occurrence of polymorphism. Second, using different online tools, the disease-causing nature of the variant was verified with tools such as MutationTaster (25), DANN (26), LRT (27), FATHMM (28), EIGEN (29), and BayesDel_addAF (30) along with American College of Medical Genetics (ACMG) guidelines (31).

#### Variant conservation

The conservation of the variant across different species was checked using the NCBI-HOMOLOGENE database (https://www.ncbinlm.nih.gov/homologene).

#### Sanger sequencing

The variant obtained was filtered and identified as disease causing using Sanger sequencing following standard methods (21). Genomic sequences of the gene were retrieved from the UCSC genome database browser (http://genome.ucsc.edu/cgi-bin/hgGateway). The primers for Sanger sequencing were designed using the PRIMER3 software (32). The exon 11 containing the identified *DNM1* variant was amplified using exon-specific primers through polymerase chain reaction (PCR) following standard protocols. Purification of the PCR-amplified DNA was performed according to the manufacturer's instructions. The data obtained from the Sanger sequencing were analyzed using BIOEDIT tool.

#### *In silico* analysis—3D protein structure modeling

The crystal structure of the DNM1 protein was retrieved from RCSB under accession number 4UUK. The preparation module of UCSF Chimera (https://www.cgl.ucsf.edu/chimera/) was used to resolve the missing residues and non-sense mutation was inserted using the mutagenesis tool PyMOL, followed by energy minimization with MMFF94 force field implemented in a molecular operating environment (MOE) (https://www.chemcomp.com/Products.htm). The mutation was induced in the structure using the mutagenesis tool implemented in PyMOL (https://pymol.org/2/) as described by Alotaibi et al. (33).

#### Modeling and structure minimizations

The crystal structure containing missing loop residues at the N terminal, middle, and C terminal were built by the loop module of Chimera; afterward, the structure was subjected to energy minimization. Then, non-sense mutation was inserted at position 468. Surprisingly upon visual inspections and comparison with the 3D structure of wild-type (WT) DNM1, we found that the novel variant undergoes structural changes in the loop regions of the respective protein.

#### Molecular dynamics simulation

Molecular dynamics simulation was carried out for the wild-type and mutant proteins to comprehend the dynamic behavior of the mutated and wild states. The protein force field ff14SB implemented in the Amber 20 package was applied (34). To solvate each system, the tip3p water model with an 8.0 box dimension was used. Each system was completely solvated and neutralized by the addition of four Na+ ions, which balanced out the charges in each system. After 6,000 steps of the steepest descent and conjugate gradient minimization technique, the density was then equilibrated for 2 ns. For another 2 ns, the entire system was then equilibrated at constant pressure. The temperature was kept constant at 300 K using the Langevin thermostat. Moreover, a 200 ns molecular dynamics (MD) was run after equilibration (33). For electrostatic interactions, the particle Mesh Ewald (PME) method was used. Hydrogen-containing covalent bonds were handled via the SHAKE algorithm. Finally, at constant pressure and temperature MD simulation was performed using PMEMD (35).

#### RT-qPCR

In order to functionally validate the variant, total RNA was extracted from all the available family members to investigate the relative mRNA *DNM1* expression using GAPDH [DQ403057] as the internal control “house-keeping” gene. cDNA was synthesized using standard methods from total RNA using standard cDNA reverse transcription kit (36). Primer-bank database (https://pga.mgh.harvard.edu/Parabiosys/) was used to design the primer pair and will be provided on request. PCR SYBRGreen Master Mix was used for the qPCR reaction and Quant-Studio 6 Flex Real-Time PCR-System was used. All the reactions were repeated independently, performed in triplicate, and data were analyzed using Expression-Suite software version 1. GAPDH was used as an endogenous control.

## Results

### Clinical report

The current study reports on a single affected individual (II-1). Parents were first-degree cousins, and the pedigree depicted an autosomal recessive pattern of inheritance (Figure 1). The family had no previous history of such disorder. The parents and two other siblings were normal. The proband revealed features such as neurodevelopmental disorder, mild microcephaly, moderate to severe ID, speech issues, seizures, epileptic encephalopathy, and hypotonia.

His growth parameters at age 9 were as follows: height: 120.6 cm [2 percentile (−2.1 SD)], weight: 22.7 kg [5 percentile (−1.68 SD)], and head circumference (HC): 48.9 cm [<1 percentile (−2.8 SD)]. All the biochemical tests were unremarkable, and no metabolic disorder was observed. Ophthalmological examination showed unremarkable results, including normal funduscopic examination. Electroencephalogram (EEG) revealed multifocal epileptic discharges, thus suggesting epileptic encephalopathy. There were no dysmorphic features observed in the affected individual. He was unable to hold his head without support, roll over, and hold objects properly. Socially, he is shy and avoids making eye contact. Speech and language development were also delayed. The detailed clinical information is mentioned in Table 1.

**Table 1:** Clinical characteristic and general information of a proband with diverse neurodevelopmental phenotypes.

Patient ID	NDD-0685
Mutation	c.1402G>T; (p.Glu468*)
Inheritance	Homozygous
Providing center/clinicians	UMT and University of Education
Year of birth	10
Age at inclusion	9
Gender	Male
Ethnicity	South Asian
Personal history	
Pregnancy and birth	Unremarkable pregnancy
Exam at birth	Not available
Height, weight, and head circumference	Height: 120.6 cm [2 percentile (−2.1 SD)] Weight: 22.7 kg [5 percentile (−1.68 SD)] HC: 48.9 [<1 percentile (−2.8 SD)] https://simulconsult.com/
Dysmorphic features	Unremarkable
Other features	Unremarkable
Development	
Development prior to epilepsy onset	Developmental delay
Development at last follow-up	Delayed (age: 9 years)
Stagnation or regression of development? If yes, age of onset	Yes, according to parents
Motor milestones	Delayed
Cognitive development, degree of ID	Mild–severe
Neurological examination	There is no intra cranial hemorrhage, territory infarction, mass effect, midline shift, or hydrocephalus. Generalized volume loss manifested by a prominent ventricles and extra-axial CSF spaces. Posterior fossa structures are grossly unremarkable. Mastoid air cells, paranasal sinus, and visualized orbits are unremarkable. There is a delay in myelination of the midline structures corresponding to the patient's age. There is no parenchymal signal intensity abnormality identified. No hydrocephalus, space-occupying lesion, or extra-axial collection. The posterior fossa structures are unremarkable. The intracranial arteries and veins are unremarkable. The visualized parts of the orbits and paranasal sinuses are unremarkable. **Conclusion**: Generalized volume loss. Delayed myelination of the midline and deep white matter structures.
Movement disorder	N/A
Behavioral/psychiatric disturbances	Aggression, urinary incontinence, personality problems, etc.
Epilepsy	
Seizure onset	1–2 years
Seizure type at onset	Tonic
Seizure outcome	Aggressive behaviors due to seizures
Fever sensitivity	Mild fever after every 6–8 months
Other provoking factors	N/A
Special features of epilepsy	N/A
EEG (provide characteristic screenshots if possible)	
EEG at onset	Unremarkable
Course of EEG	Unremarkable
Treatment	
Overall antiepileptic drug response	Decrease in seizure frequency and aggressive behaviors according to parents
Drugs with positive response	N/A
Drugs with no response or provocation	N/A
Special therapeutic strategies (KD, VNS, other)	None
Diagnostics	
MRI	Abnormal
Other genetic abnormalities	
Family history	None
Investigations (panel, exome sequencing, single gene sequencing?)	Exome sequencing

KD, ketogenic diet; VNS, vagus nerve stimulation.

### Brain MRI

**Figure 2:**
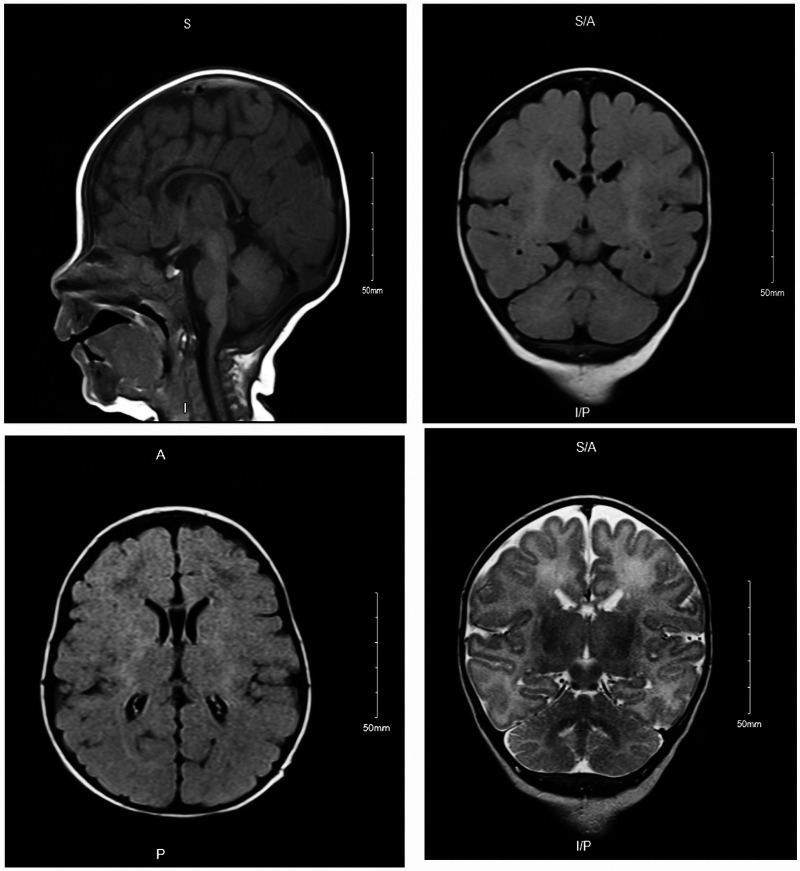
Brain MRI of affected individual (IV-2) revealed generalized volume loss manifested by a prominent ventricles and extra-axial CSF spaces. Posterior fossa structures, paranasal sinus, mastoid air cells, and visualized orbits were unremarkable.

The brain MRI revealed no territory infarction, intra cranial hemorrhage, mass effect, hydrocephalus, or midline shift. A generalized volume loss was observed manifested by a prominent ventricles and extra-axial cerebrospinal fluid (CSF) spaces (Figure 2). Posterior fossa structures showed unremarkable presentation. Paranasal sinus, mastoid air cells, and visualized orbits were unremarkable. A delay in myelination of the midline structures was observed, corresponding to the patient's age. No abnormality associated with parenchymal signal intensity was identified. No extra-axial collections, hydrocephalus, and/or space-occupying lesions were observed. The posterior fossa structures, intracranial arteries, and veins were unremarkable. The paranasal sinuses and visualized parts of the orbits were unremarkable.

### Molecular genetic analysis

Chromosomal and cytogenetic analysis did not reveal any disease-causing genomic alterations that might be the cause of the observed phenotype in the proband. The DNA of the affected individual (II-2) was subjected to WES using standard methods. WES filtration data analysis revealed a novel homozygous non-sense mutation (c.1402G>T; p.Glu468*) in the exon 11 of the *DNM1* (NM_004408.4; 9q34.11; ENST000003729) gene. The novelty of the variant was checked in the online literature and HGMD database 2022. The variant results in a premature stop-codon at 468 amino acid position. The mutation might result in a shorter protein that might result in non-sense-mediated decay (NMD).

### Sanger sequencing

**Figure 3:**
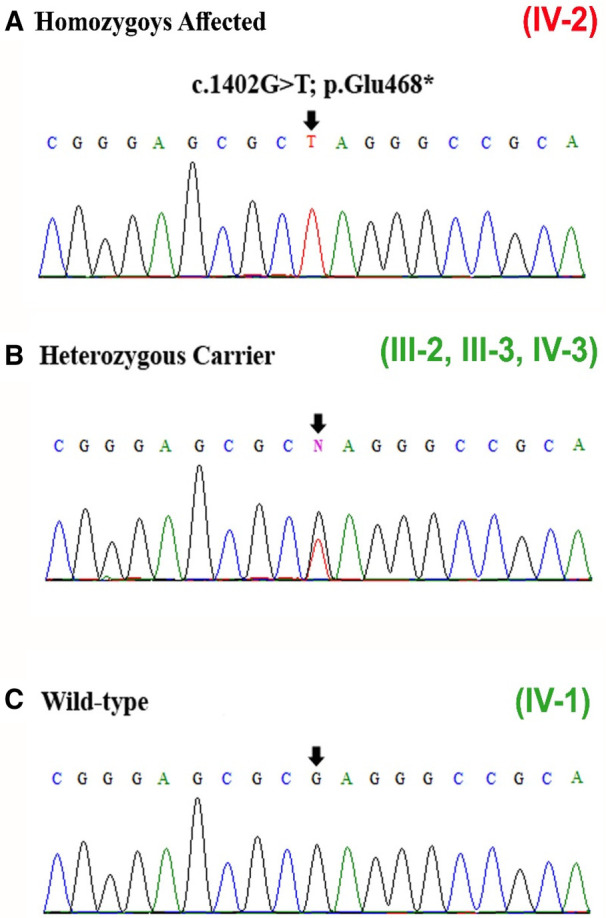
(**A–C**) Sanger sequencing results of exon 11 of the *DNM1* gene showing the homozygous variant [c.1402G>T] identified in the patient (IV-2), carriers (heterozygous) (III-2, III-3, IV-3), and wild type (IV-1). The site of mutation is indicated by black arrows.

To consider the segregation of the variant with the disease phenotypes, the identified variant [c.1402G>T] was Sanger sequenced in all family members, which segregated perfectly within the family. The variant was observed in homozygous state in the proband, both the parents (III-2, III-3) and sibling (brother; IV-3) were carriers, and the sibling (brother; IV-1) was wild type (Figures 3A–C).

### Pathogenicity and conservation

**Figure 4:**
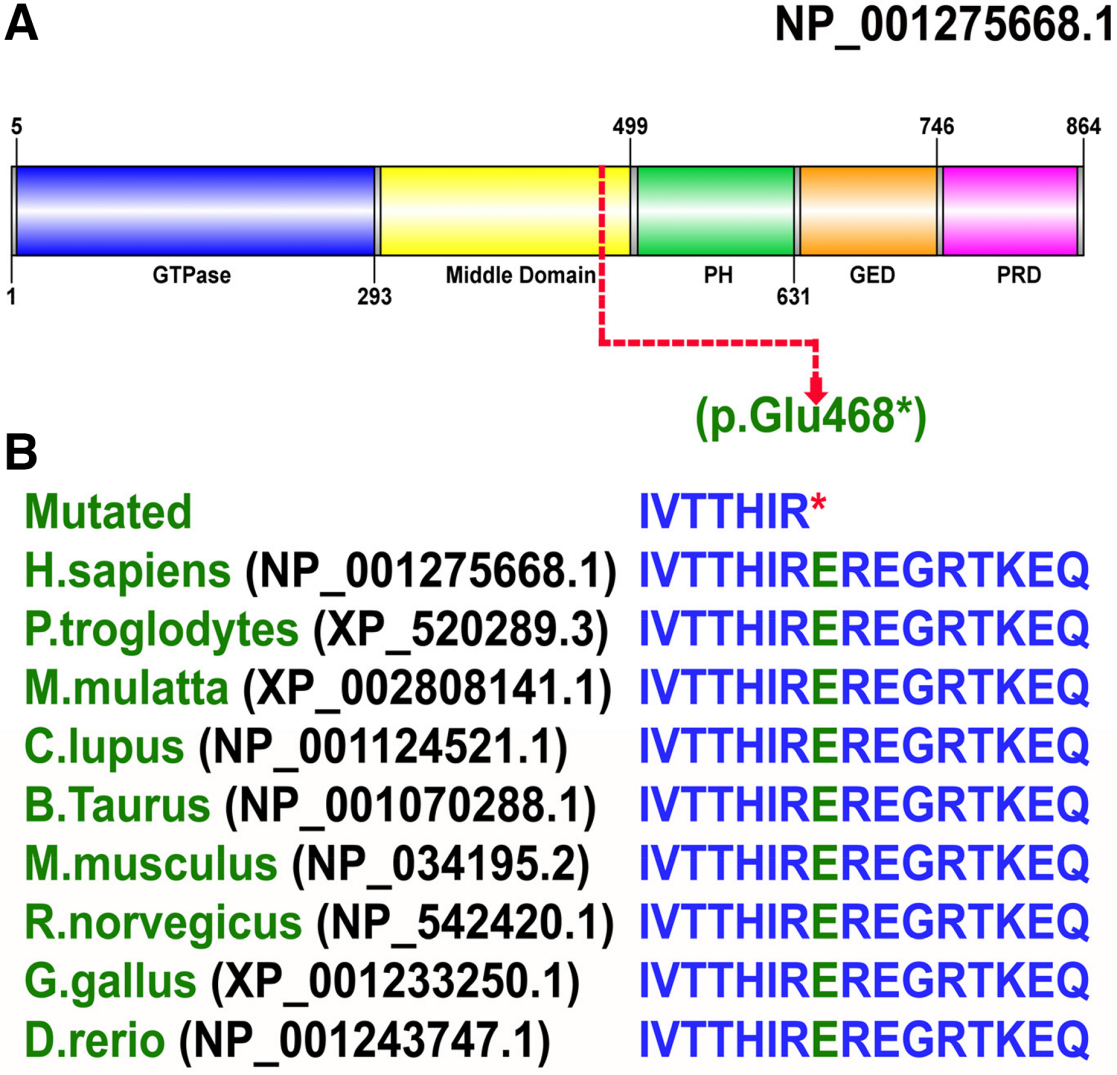
(**A**) Schematic representation of the DNM1 protein organized into five domains: (a) an N-terminal GTPase domain (5-293 aa); (b) a middle domain (300-499 aa); (c) a PH domain (505-631 aa); (d) a GED (635-746 aa), and (e) a PRD (750-850 aa). The site of mutation p. Glu468* shown in red is in the middle domain (300–499 aa). (**B**) Conservation of Glu at amino acid 468 in DNM1 in species with a known DNM1 orthologs.

The identified non-sense variant (p. Glu468*) was classified as disease causing using different online prediction tools such as MutationTaster (disease-causing [1]), DANN (disease-causing [0.997]), LRT (pathogenic [0]), FATHMM (pathogenic [0.9844]), EIGEN (pathogenic [1.130]), and BayesDel_addAF (pathogenic [0.5774]), and according to the ACMG guidelines, the identified variant was classified as pathogenic (Class I). According to gnomAD, the variant was not observed in homozygous state in the public database thus suggesting a non-polymorphic nature of the variant. Furthermore, the Glu amino acid at position 468 is highly conserved across several species (Figures 4A,B), suggesting key role of this variant in the neurodevelopment.

### *In silico* analysis—3D protein modeling

**Figure 5:**
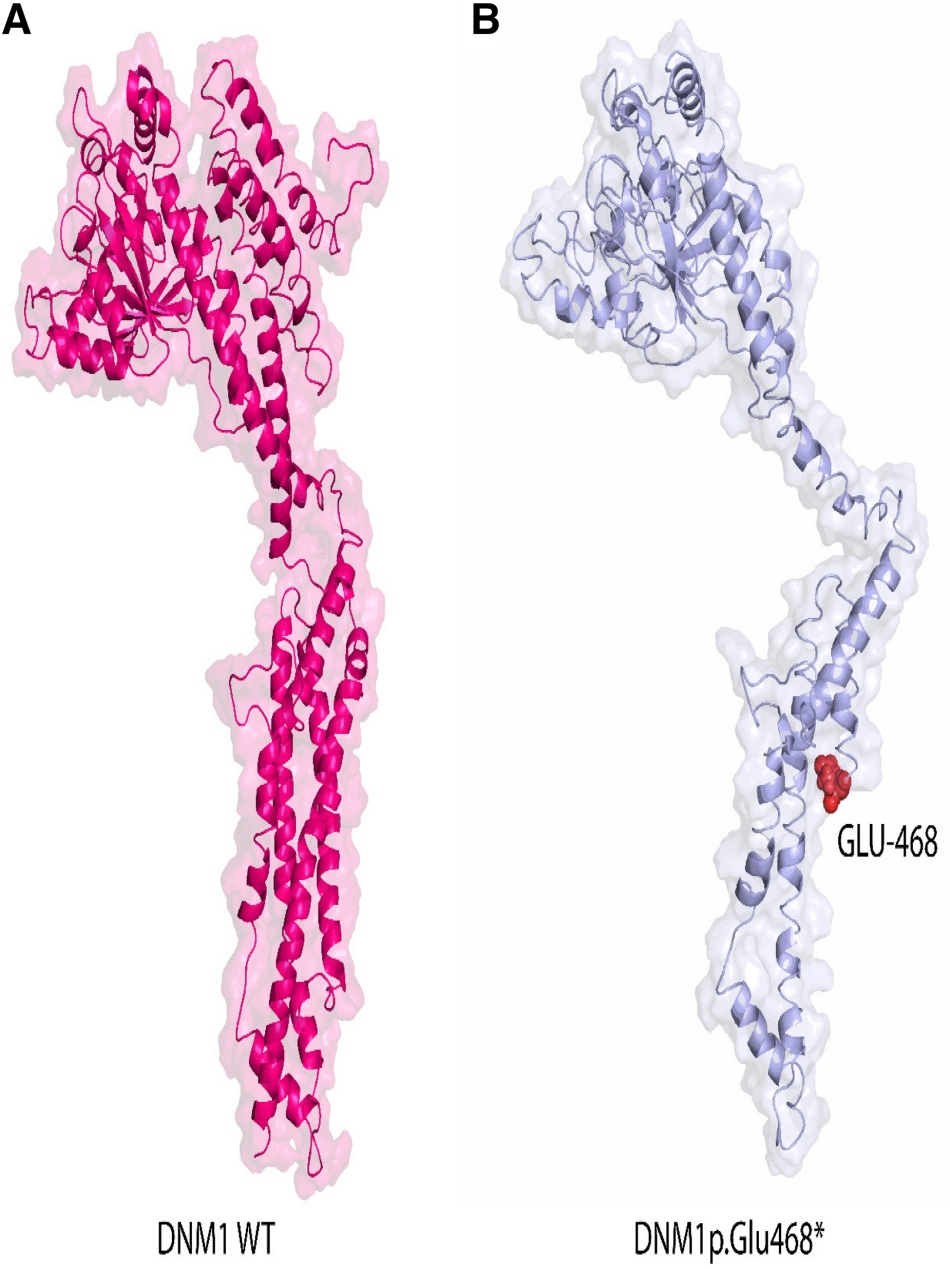
Representation of the 3D structural model of human wild-type DNM1^(wt)^ protein (pink) and mutated protein (light blue). Red color indicates position of novel mutation Glu at position 468 in DNM1^p.Glu468*^ protein identified in this study. *Accession number* NP_001275668.1 version was used.

Then non-sense mutation was inserted at position 468 as shown in Figure 4A. Remarkably upon visual inspections and comparison with the 3D structure of wild-type DNM1, we found that the novel variant undergoes structural changes in loop regions of the respective proteins. If the DNM1 protein is not degraded in the NMD, these changes in the final protein structure might lead to a small non-functional protein that might not perform proper function and downstream signaling (Figures 5A,B).

### RMSD analysis

**Figure 6:**
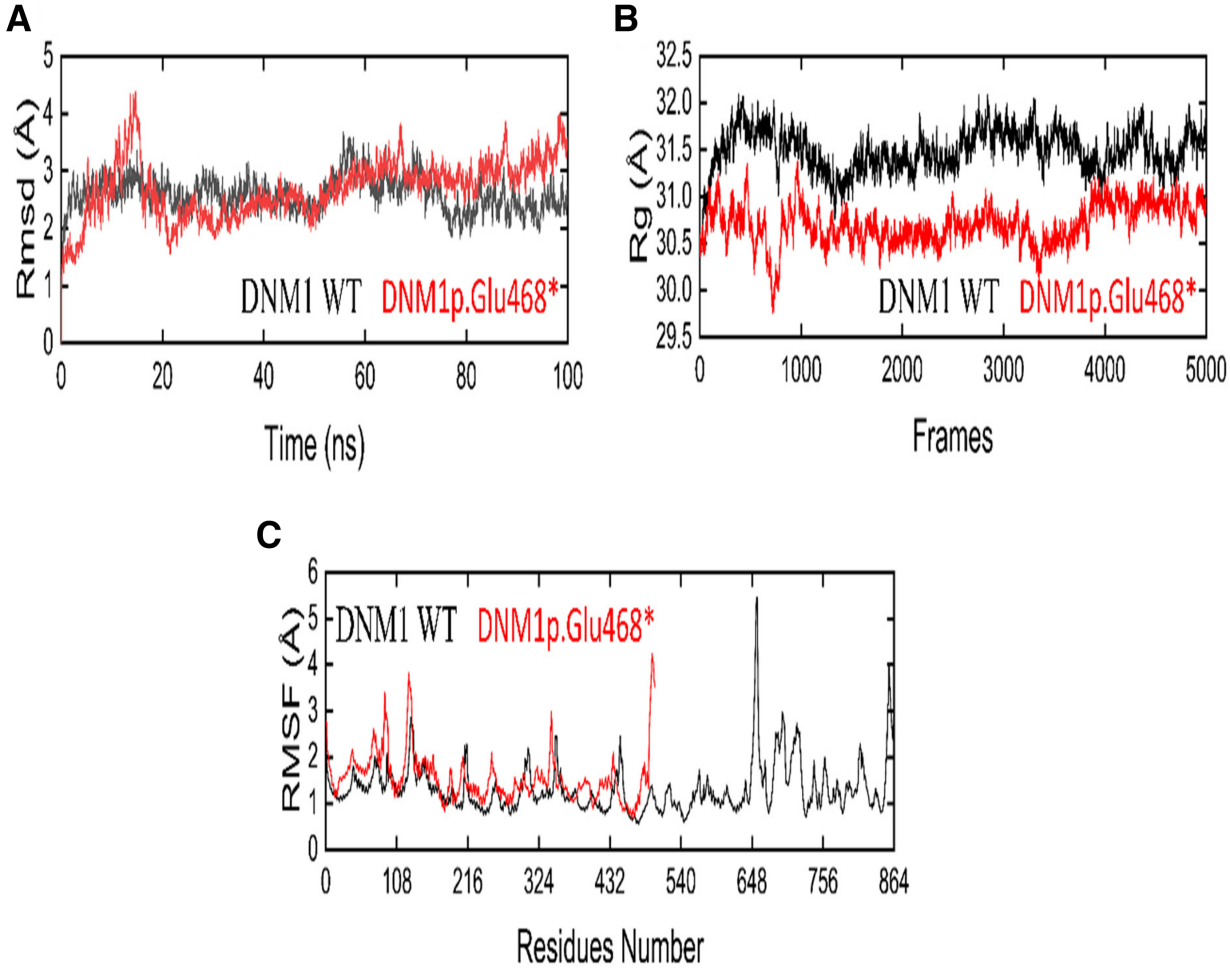
The dynamic behavior of wild-type (WT) and DNM1 ^(p. Glu468*)^ mutant variant. (**A**) RMSD of wild-type DNM1 and DNM1 ^(p. Glu468*)^. (**B**) Radius of gyration for wild-type DNM1 and DNM1^(p. Glu468*)^. (**C**) RMSF of wild-type DNM1 and DNM1^(p. Glu468*)^, respectively.

Root mean square deviation (RMSD) analysis showed that the value of mutant increased rapidly to 4.5 Å from 10 to 15 ns but then decreased to 2 Å for 50 ns. After 50 ns, RMSD slightly increased to 2.5 Å and minor deviations were seen during 65–68 and 85–90 ns, but overall, the structure showed a stable behavior till 100 ns MD run. On the other hand, the wild-type DNM1 revealed a stable behavior during the entire 100 ns MD simulation, only minor deviations were seen during 55–75 ns and the DNM1^Wt^ protein RMSD value of 2.8 Å was attained, and throughout the simulation, it revealed stability. RMSD plots of both the wild-type and the mutated proteins are shown in Figure 6A.

### Rg analysis

By analyzing the structural compactness along with the stability of the folded or unfolded protein, the radius of gyration (Rg) calculation was carried out. For the mutant and wild states, the average values of Rg were in the range of 29.5–31.0 and 31.0–32.0, respectively. It is clear from the Rg calculation that the wild-type system revealed more compactness as compared to the mutant form during the MD simulation. The Rg plots for both the wild and mutant states are shown in Figure 6B.

### RMSF analysis

The root mean square fluctuation (RMSF) was calculated for the C-alpha atoms of both the wild and mutant states in order to explore the fluctuating residues during the 100 ns MD simulation. In the mutant state, the residues 105–107, 110–113, and residue 325 showed more fluctuations during the MD simulation while the residues 114–324 showed stability. On the other hand, the wild state showed major fluctuations at residues 648–652 and the residues 740–750 showed minor fluctuations, while all other residues showed stability during the entire 100 ns MD run. Figure 6C displays the RMSF plots for the wild-type as well as mutant protein.

### Non-sense variant in *DNM1* reduced mRNA expression

**Figure 7:**
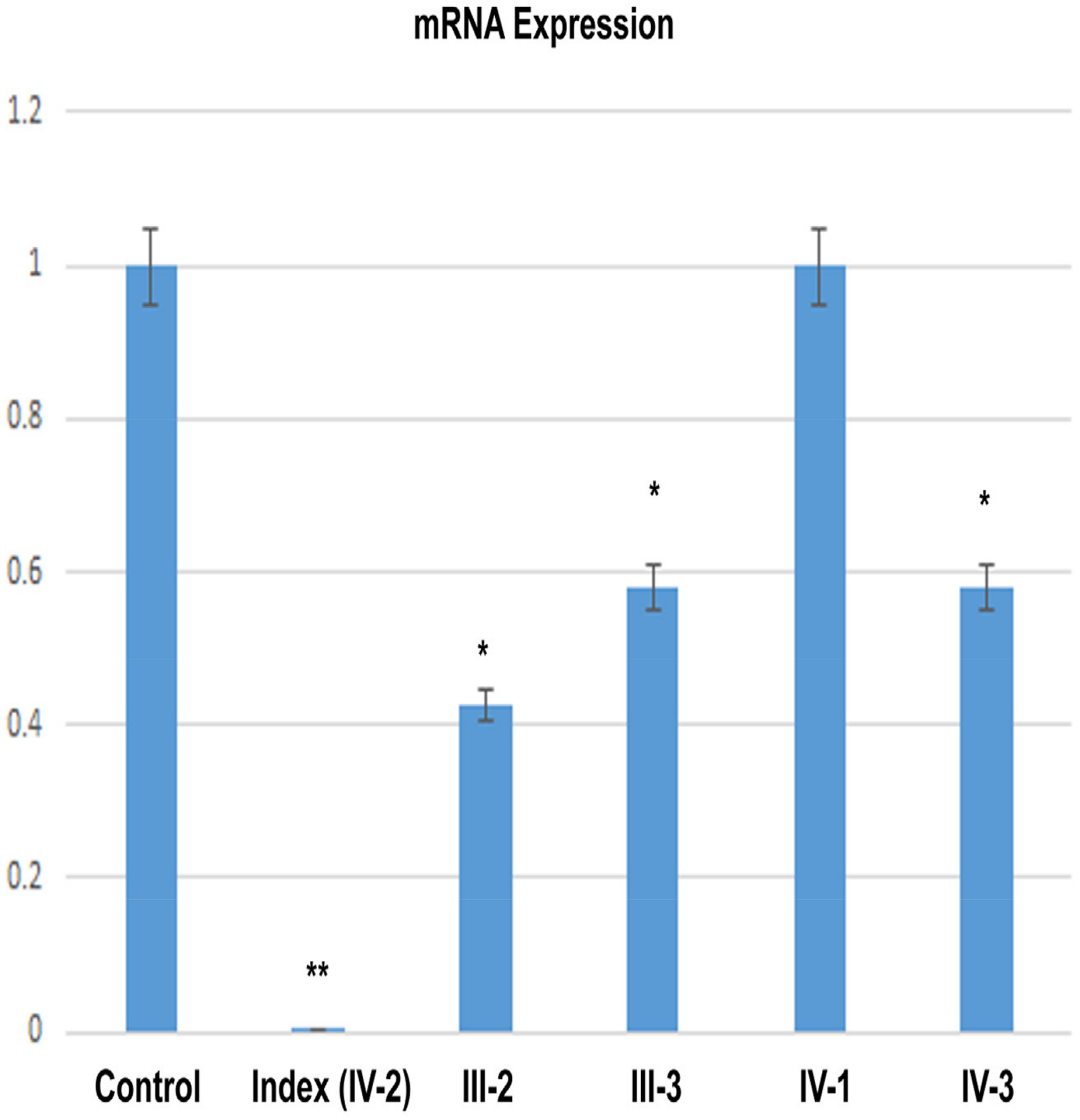
RT-qPCR shows a significant decrease of the *DNM1* mRNA expression in an index patient (IV-2) in comparison to control or other members of the family (III-2, III-3, IV-1, and IV-3).

The relative expression data of *DNM1* gene in the affected individual, parents, and normal control individuals showed that the proband (IV-2) having the disease-causing homozygous variant (p. Glu468*) had substantial reduction in the *DNM1* gene expression as compared to the wild type (control) and carrier (parents) (Figure 7).

## Discussion

Disorders associated with *DNM1* pathogenesis have a unique clinical appearance. Patients having disease-causing variants in the *DNM1* gene typically reveal profound hypotonia from birth, infantile spasms that might lead to the Lennox–Gastaut syndrome, developmental delay, microcephaly (few patients), cortical visual impairment, speech delay, and intellectual disability (11, 12).

To date, 46 patients with epileptic encephalopathy and *DNM1* pathogenic variants have been described in 45 families (37–41).

*DNM1* gene encodes dynamin-1, a protein required for clathrin-mediated endocytosis and synaptic vesicle recycling. The fission of clathrin-coated vesicles from the plasma membrane by dynamin-1 oligomers promotes vesicle-mediated neurotransmitter release at the synapse (14). It is only found in neurons in the presynaptic terminal, where it participates in synaptic vesicle endocytosis and membrane recycling after neurotransmitter release (14). In mammals, this gene can produce various isoforms, which are characterized primarily by alternative splicing of the tenth exon—some transcripts have exon 10b, while others contain exon 10c (42).

Herein, we describe a proband having pathogenic Class-I variant in the *DNM1* that revealed neurodevelopmental features such as developmental delay, moderate–severe ID, microcephaly, hypotonia, seizures, and speech issues. Features reported previously, such as visual impairment, EEG abnormality, and scoliosis, were not observed in the patient reported here (Table 1). Interestingly, Yigit et al. (41) reported the only two affected individuals having homozygous *DNM1* variants [c.97C>T; p.(Gln33*), c.850C>T; p.(Gln284*)] that were having a non-sense mutation and revealed hypotonia, spasticity, dystonia, and feeding abnormalities that overlapped with our patient features (41). However, scoliosis and visual impairment were not observed in our patient.

Previously, only *de novo* variants in the *DNM1* were linked to developmental and epileptic encephalopathy (Table 2; MIM 620352, MIM 616346). Clinically, affected individuals having *DNM1* heterozygous missense variants show phenotypic heterogeneity with early-onset epilepsy, hypotonia, and developmental delay (11). These patients have normal MRI analysis with cerebral volume loss over time. Five patients with early-onset DEE (DEE31, OMIM 616246) were the first to have heterozygous missense mutations in the *DNM1* gene (13). Since then, many patients having heterozygous, primarily *de novo DNM1* mutations, have been reported in the literature (Table 2). Except for one *de novo* in-frame 06 bp insertion, all observed variants disrupt highly conserved residues and are projected to have a dominant-negative impact on the dynamin-1 function. Detailed clinical comparison of all the patients reported with *DNM1* variants have been summarized in Table 3.

**Table 2:** Mutations reported in DNM1 associated with neurodevelopmental disorders.

Mutation type	Amino acid change	cDNA change	Associated phenotype
Missense	p.Leu16Met	c.46C>A	Nicotine dependence risk, association with
Non-sense	p.Gln33*	c.97C>T	Developmental and epileptic encephalopathy
Missense	p.Gly38Ser	c.112G>A	Seizures and psychomotor delay
Missense	p.Gly43Asp	c.128G>A	Epileptic encephalopathy, early infantile
Missense	p.Gly43Ser	c.127G>A	Lennox–Gastaut syndrome
Missense	p.Ser45Arg	c.135C>A	Epileptic encephalopathy
Missense	p.Ser45Asn	c.134G>A	Encephalopathy
Missense	p.Val47Met	c.139G>A	Moderate intellectual disability, speech delay, seizures macrocephaly, and hypotonia
Missense	p.Thr65Asn	c.194C>A	Epileptic encephalopathy
Missense	p.Arg67Cys	c.199C>T	Neurodevelopmental disorder
Missense	p.His85Gln	c.255C>A	Epilepsy, childhood
Non-sense	p.Ser126*	c.377C>A	Nicotine dependence risk
Missense	p.Gly139Arg	c.415G>A	Developmental disorder
Missense	p.Gly139Val	c.416G>T	Epileptic encephalopathy with infantile spasms
Missense	p.Pro144Leu	c.431C>T	Epileptic encephalopathy
Missense	p.Gln148Arg	c.443A>G	Epileptic encephalopathy
Missense	p.Gln148Lys	c.442C>A	Epilepsy with/without neurodevelopmental delay
	p.Ala177Pro	c.529G>C	Epileptic encephalopathy
Missense	p.Asn178Lys	c.534C>G	GLUT1 deficiency syndrome
Missense	p.Arg199Leu	c.596G>T	Neurodevelopmental disorder
Missense	p.Thr200Ile	c.599C>T	Intellectual disability and epilepsy
Missense	p.Lys206Asn	c.618G>C	Epileptic encephalopathy
Missense	p.Lys206Glu	c.616A>G	Epileptic encephalopathy with infantile spasms
Missense	p.Asp211Val	c.632A>T	Developmental and epileptic encephalopathy
Missense	p.Arg228Leu	c.683G>T	Nicotine dependence risk, association with
Non-sense	p.Tyr231*	c.693C>A	Nicotine dependence risk, association with
Missense	p.Arg237Trp	c.709C>T	Epileptic encephalopathy
	p.Ser238Ile	c.713G>T	Encephalopathy with seizures
Non-sense	p.Arg256*	c.766C>T	Neurodevelopmental disorder
Missense	p.Pro263Leu	c.788C>T	Neurodevelopmental disorder
Missense	p.Arg266Cys	c.796C>T	Progressive bilateral mesial temporal sclerosis
Missense	p.Arg271His	c.812G>A	Neurodevelopmental disorder
Non-sense	p.Gln284*	c.850C>T	Developmental and epileptic encephalopathy
Missense	p.Ile289Phe	c.865A>T	Epileptic encephalopathy, early onset
	p.Arg297Trp	c.889C>T	Neurodevelopmental disorder
Missense	p.Phe336Phe	c.1008C>T	Nicotine dependence, association with
Missense	p.Gly346Asp	c.1037G>A	Neurodevelopmental disorder
Missense	p.Gly346Val	c.1037G>T	Epileptic encephalopathy with infantile spasms
Missense	p.Gly359Ala	c.1076G>C	Lennox–Gastaut syndrome
Missense	p.Gly359Arg	c.1075G>A	Epileptic encephalopathy with infantile spasms
Missense	p.Gly359Arg	c.1075G>C	Epileptic encephalopathy, early onset
Missense	p.Gly359Glu	c.1076G>A	Epileptic encephalopathy, early infantile
Missense	p.Glu373Lys	c.1117G>A	Encephalopathy with seizures
Missense	p.Arg385Gln	c.1154G>A	Neurodevelopmental disorder
	p.Tyr390Asp	c.1168T>G	Developmental and epileptic encephalopathy
Missense	p.His396Asp	c.1186C>G	Developmental disorder
Missense	p.Gly397Asp	c.1190G>A	Epileptic encephalopathy with infantile spasms
Missense	p.Arg399Gly	c.1195A>G	Infantile spasms
Missense	p.Pro405Leu	c.1214C>T	Developmental and epileptic encephalopathy
Non-sense	p.Arg421*	c.1261C>T	Neurodevelopmental disorder
Missense	p.Pro423Leu	c.1268C>T	Neurodevelopmental disorder
Missense	p.Val431Ile	c.1291G>A	Neurodevelopmental disorder
Missense	p.Glu434Lys	c.1300G>A	Neurodevelopmental disorder
Missense	p.Gln448His	c.1344G>C	West syndrome
Missense	p.Arg465Gln	c.1394G>A	Neurodevelopmental disorder
Missense	p.Ile479Ile	c.1437C>T	Developmental disorder
Missense	p.Ile533Thr	c.1598T>C	Neurological disease
Missense	p.Lys535Glu	c.1603A>G	Developmental delay and autistic symptoms
Missense	p.Lys539Glu	c.1615A>G	Epilepsy
Non-sense	p.Lys554*	c.1660A>T	Neurodevelopmental disorder
Missense	p.Arg724Trp	c.2170C>T	Epilepsy
Splice site		c.11978G>A	Intellectual disability
Splice site		c.1335 + 1701C>T	Neurodevelopmental disorder
Small deletion	p.(Pro117Argfs*14)	c.350delC	Facial dysmorphism, developmental delay, seizures, and nystagmus
Small deletion	p.(Gly359Glufs*55)	c.1071delA	Autism
Small deletion	p.(Lys694Argfs*21)	c.2079delC	Intellectual disability with epilepsy
Small insertion	p.(Gln155_Ile156insMet)	c.465_467dupGAT	Epileptic encephalopathy, early infantile
Small insertion	p.(Asn363_Arg364insLeuPro)	c.1090_1091insTTCCAC	Encephalopathy
Small insertion	p.(Tyr541*)	c.1622dupA	Neurodevelopmental disorder

*Nonsense mutation.

**Table 3:** Clinical comparison of patients reported with DNM1 mutation.

Reference	EuroEPINOMICS-RES Consortium et al. (43)					Nakashima et al. (17)		Brereton et al. (18)		Yigit et al. (41)	
	c.529G>C;	c.618G>C;	c.1076G>C;	c.709C>T;	c.194C>A;	c.127G>A;	c.709C>T;	c.1603A>G;	c.1603A>G;	c.97C>T;	c.850C>T;
DNM1 variant	p.Ala177Pro	p.Lys206Asn	p.Gly359Ala	p.Arg237Trp	p.Thr65Asn	p.Gly43Ser	p.Arg237Trp	p.Lys535Glu	p.Lys535Glu	p.(Gln33*)	p.(Gln284*)
Zygosity	Het	Het	Het	Het	Het	Het	Het	Het	Het	Hom	Hom
Number of patients	1	1	1	1	1	1	1	1	1	1	1
Age at last follow-up	15 years	8 years	6 years	13 years	6 years	15 years	6 years	8 years, monozygotic twins		5 years	3 years 8 m
Somatic growth											
Weight at follow-up	n.r.	n.r.	n.r.	n.r.	n.r.	n.r.	n.r.	24th centile	30th centile	−2.1 SD	−3.7 SD
Length at follow-up	n.r.	n.r.	n.r.	n.r.	n.r.	n.r.	n.r.	28th centile	22th centile	−2.4 SD	−3.1 SD
HC at follow-up	n.r.	n.r.	n.r.	Microcephaly	n.r.	n.r.	n.r.	85th centile	95th centile	−4.4 SD	−4.5 SD
Motor and speech development											
Sitting	Yes	Yes	Yes	No	No	Yes	Yes	Yes	Yes	No	No
Walking	Yes	No	No	No	No	Yes	Yes	Yes	Yes	No	No
Functional hand use	n.r.	n.r.	n.r.	n.r.	n.r.	n.r.	n.r.	Yes	Yes	No	No
Verbal expression	No	No	No	No	No	No	No	Yes	Yes	No	No
Neurological features											
Epilepsy (onset; type)	7 m; VS	6 m; VS	2 m; IS	12 m; VS	13 m; VS	11 m; LGS	10 m; West syn.	No	No	4 m; FS	6 m; FS
EEG	MFED, GSW, slow bg	Hyps, MFED, GSW, slow bg		Hyps, GSW, slow bg	Hyps, MFED, slow bg	Partial hyps, MFED					
			GSW				MFED, GSW	Normal	Normal	Hyps, MFED	Hyps, MFED
Hypotonia	Yes	Yes	Yes	Yes	Yes	n.r.	Yes	Yes	Yes	Yes	Yes
Spasticity	n.r.	n.r.	n.r.	n.r.	n.r.	n.r.	n.r.	n.r.	n.r.	Yes	No
Dystonia	n.r.	n.r.	n.r.	n.r.	n.r.	n.r.	Yes	n.r.	n.r.	Yes	Yes
Feeding problems	n.r.	n.r.	n.r.	n.r.	n.r.	n.r.	n.r.	n.r.	n.r.	Yes	Yes
Visual impairment	n.r.	n.r.	n.r.	Yes	Yes	n.r.	n.r.	n.r.	n.r.	Yes	Yes
Miscellaneous	n.r.	n.r.	Seizure free on ketogenic diet	n.r.	n.r.	n.r.	Arachnoid cyst right temporal lobe	Mild-moderate ID, autism		Scoliosis	Mild bilat. optic atrophy
											
											
Neuroimaging	Normal	Normal	Normal	Cerebral atrophy	Cerebral atrophy	Normal	n.r.	n.p.	Normal	Normal	Cerebral atrophy

Reference	von Spiczak et al. (11)														Present study
	c.127G>A;	c.134G>A;	c.194C>A;	c.416G>T;	c.529G>C;	c.616A>G;	c.618G>C;	c.709C>T;	c.731 G>A;	c.1037G>T;	c.1075G>A;	c.1076G>C;	c.1117G>A;	c.1109G>A;	c.1402G>T
DNM1 variant	p.Gly43Ser	p.Ser45Asn	p.Thr65Asn	p.Gly139Val	p.Ala177Pro	p.Lys206Glu	p.Lys206Asn	p.Arg237Trp	p.Ser238Ile	p.Gly346Val	p.Gly359Arg	p.Gly359Ala	p.Glu373Lys	p.Gly397Asp	p.Glu468*
Zygosity	Het	Het	Het	Het	Het	Het	Het	Het	Het	Het	Het	Het	Het	Het	Hom
Number of patients	1	1	1	1	1	1	1	7	1	1	2	1	1	1	1
Age at last follow-up	8 years	2 years	8 years	18 years	15 years	9 years	8 years	2–24 years	19 years	13 years	both 1 year	7 years	5 years	2 years	9 years
Somatic growth															
Weight at follow-up	n.r.	n.r.	n.r.	n.r.	n.r.	n.r.	n.r.	n.r.	n.r.	n.r.	n.r.	n.r.	n.r.	n.r.	22.7 kg [5 percentile (−1.68 SD)]
Length at follow-up	n.r.	n.r.	n.r.	n.r.	n.r.	n.r.	n.r.	n.r.	n.r.	n.r.	n.r.	n.r.	n.r.	n.r.	2 percentile (−2.1 SD)
HC at follow-up	Microcephaly	n.r.	n.r.	n.r.	n.r.	n.r.	n.r.	n.r.	n.r.	n.r.	Microcephaly	Microcephaly	n.r.	Microcephaly	48.9 cm [<1 percentile (−2.8 SD)] microcephaly
Motor and speech development															
Sitting	No	No	No	No	No	No	No	No	No	No	No	No	No	No	Delayed
Walking	No	No	No	No	No	No	No	No	No	No	No	No	No	No	Delayed
Functional hand use	n.r.	n.r.	n.r.	n.r.	n.r.	n.r.	n.r.	n.r.	n.r.	n.r.	n.r.	n.r.	n.r.	n.r.	Delayed
Verbal expression	No	No	No	No	No	No	No	No	No	No	No	No	No	No	No
Neurological features															
Neurological features															
Epilepsy (onset; type)	3 weeks; AS, MS	No	13 m; VS	4 m; VS	7 m; VS	2 m; VS	6 m; VS	3–12 m; VS	8 m; GTCS	6 m; VS	No/1 m, VS	2 m; IS	4 y 6 m; VS	3 m; MS	4 years
			Hyps, MFED, slow bg	Hyps, MFED, slow bg			Hyps, MFED, slow bg	Hyps, MFED,		Hyps, MFED, slow bg				Hyps, MFED, slow bg	Unremarkable
EEG	Slow bg	Normal			MFED, slow bg	MFED		Slow bg in most pat.	n.r.		MFED, slow bg	SSW	GSW, slow bg		
Hypotonia	Yes	Yes	Yes	Yes	Yes	Yes	Yes	Yes	Yes	Yes	Yes	Yes	n.r.	Yes	Yes
Spasticity	Yes	n.r.	n.r.	Yes	n.r.	n.r.	n.r.	Yes	n.r.	n.r.	n.r./yes	n.r.	n.r.	n.r.	Yes
Dystonia	Yes	n.r.	n.r.	Yes	n.r.	Yes	n.r.	Yes	n.r.	n.r.	n.r./yes	n.r.	n.r.	n.r.	Yes
Feeding problems	n.r.	n.r.	n.r.	n.r.	n.r.	n.r.	n.r.	n.r.	n.r.	n.r.	n.r.	n.r.	n.r.	n.r.	Yes
Visual impairment	n.r.	n.r.	n.r.	n.r.	n.r.	n.r.	n.r.	n.r.	n.r.	n.r.	n.r.	n.r.	n.r.	n.r.	No
Miscellaneous	n.r.	n.r.	n.r.	n.r.	n.r.	n.r.	n.r.	n.r.	n.r.	n.r.	multifocal subcortical	n.r.		n.r.	
											Myoclonus		n.r.		Moderate–severe ID
	Normal	n.r.	Cerebral atrophy	Normal	Normal	n.r.	Normal		Normal		Thin CC/normal	Cerebral atrophy	Normal	n.r.	
Neuroimaging								Normal/cerebral atrophy		Hypoplasia of frontal lobes					Generalized volume loss

VS, visual seizure; FS, focal seizure; AS, atonic seizures; MS, myoclonic seizure; GTCS, generalized tonic-clonic seizure; IS, infantile spasms; LGS, Lennox-Gastaut syndrome; MFED, multifocal epileptiform discharges; Hyps, hypsarrhythmia; GSW, generalized spike-wave or poly spike-wave discharges; n.r., Not reported.

**Table:** 

Reference	von Spiczak et al. (11)														Present study
	c.127G>A;	c.134G>A;	c.194C>A;	c.416G>T;	c.529G>C;	c.616A>G;	c.618G>C;	c.709C>T;	c.731 G>A;	c.1037G>T;	c.1075G>A;	c.1076G>C;	c.1117G>A;	c.1109G>A;	**c.1402G>T**
DNM1 variant	p.Gly43Ser	p.Ser45Asn	p.Thr65Asn	p.Gly139Val	p.Ala177Pro	p.Lys206Glu	p.Lys206Asn	p.Arg237Trp	p.Ser238Ile	p.Gly346Val	p.Gly359Arg	p.Gly359Ala	p.Glu373Lys	p.Gly397Asp	p.Glu468*
Zygosity	Het	Het	Het	Het	Het	Het	Het	Het	Het	Het	Het	Het	Het	Het	**Hom**
Number of patients	1	1	1	1	1	1	1	7	1	1	2	1	1	1	**1**
Age at last follow-up	8 years	2 years	8 years	18 years	15 years	9 years	8 years	2–24 years	19 years	13 years	both 1 year	7 years	5 years	2 years	**9 years**
Somatic growth															
Weight at follow-up	n.r.	n.r.	n.r.	n.r.	n.r.	n.r.	n.r.	n.r.	n.r.	n.r.	n.r.	n.r.	n.r.	n.r.	22.7 kg [5 percentile (−1.68 SD)]
Length at follow-up	n.r.	n.r.	n.r.	n.r.	n.r.	n.r.	n.r.	n.r.	n.r.	n.r.	n.r.	n.r.	n.r.	n.r.	2 percentile (−2.1 SD)
HC at follow-up	Microcephaly	n.r.	n.r.	n.r.	n.r.	n.r.	n.r.	n.r.	n.r.	n.r.	Microcephaly	Microcephaly	n.r.	Microcephaly	48.9 cm [<1 percentile (−2.8 SD)] microcephaly
Motor and speech development															
Sitting	No	No	No	No	No	No	No	No	No	No	No	No	No	No	Delayed
Walking	No	No	No	No	No	No	No	No	No	No	No	No	No	No	Delayed
Functional hand use	n.r.	n.r.	n.r.	n.r.	n.r.	n.r.	n.r.	n.r.	n.r.	n.r.	n.r.	n.r.	n.r.	n.r.	Delayed
Verbal expression	No	No	No	No	No	No	No	No	No	No	No	No	No	No	No
Neurological features															Dbfbfb
Epilepsy (onset; type)	3 weeks; AS, MS	No	13 m; VS	4 m; VS	7 m; VS	2 m; VS	6 m; VS	3–12 m; VS	8 m; GTCS	6 m; VS	No/1 m, VS	2 m; IS	4 y 6 m; VS	3 m; IS, MS	4 years
			Hyps, MFED, slow bg	Hyps, MFED, slow bg			Hyps, MFED, slow bg	Hyps, MFED,		Hyps, MFED, slow bg				Hyps, MFED, slow bg	Unremarkable
	Slow bg	Normal			MFED, slow bg	MFED		Slow bg in most pat.	n.r.		MFED, slow bg	SSW	GSW, slow bg		
Hypotonia	Yes	Yes	Yes	Yes	Yes	Yes	Yes	Yes	Yes	Yes	Yes	Yes	n.r.	Yes	Yes
Spasticity	Yes	n.r.	n.r.	Yes	n.r.	n.r.	n.r.	Yes	n.r.	n.r.	n.r./yes	n.r.	n.r.	n.r.	Yes
Dystonia	Yes	n.r.	n.r.	Yes	n.r.	Yes	n.r.	Yes	n.r.	n.r.	n.r./yes	n.r.	n.r.	n.r.	Yes
Feeding problems	n.r.	n.r.	n.r.	n.r.	n.r.	n.r.	n.r.	n.r.	n.r.	n.r.	n.r.	n.r.	n.r.	n.r.	Yes
Visual impairment	n.r.	n.r.	n.r.	n.r.	n.r.	n.r.	n.r.	n.r.	n.r.	n.r.	n.r.	n.r.	n.r.	n.r.	No
Miscellaneous	n.r.	n.r.	n.r.	n.r.	n.r.	n.r.	n.r.	n.r.	n.r.	n.r.	Miscellaneous	n.r.		n.r.	
											Myoclonus		FIRES at 4.5 years		Moderate–severe ID
	Normal	n. p.	Cerebral atrophy	Normal	Normal	n. p.	Normal		Normal		Thin CC/normal	Cerebral atrophy	Normal	n. p.	
Neuroimaging								Normal/cerebral atrophy		Hypoplasia of frontal lobes					Generalized volume loss

To date, only 69 variants have been reported in the *DNM1* gene including two splice-site variants, three small deletions, three small insertions, seven non-sense, and 54 missense variants (Table 2). All missense variants associated with epileptic encephalopathy cluster in the two major functional domains of the DNM1 protein, the GTPase domain, and the middle domain. In addition, homozygous loss-of-function pathogenic variants in DNM1 have been reported to cause a severe autosomal recessive developmental and epileptic encephalopathy in three unrelated patients (37, 41). Recurrent *DNM1 de novo* splice-site variant has also been associated with developmental and epileptic encephalopathy with a dominant-negative mechanism (44). The p.Arg237Trp mutation has been observed in approximately one-third of patients. The non-sense variant identified in the present study affects a highly conserved Glu residue within the middle domain that plays a vital role in binding different targets and helps in DNM1 multimerization.

Molecular dynamics simulation is a useful tool to assess the genuine behaviors of biological molecules and their surroundings (19). In the present study, we performed MD simulations on two systems, native and mutant DNM1^p.Glu468*^. We considered parameters such as RMSD, RMSF, and Rg between the native and mutant ^p.Glu468*^ DNM1 protein structures. In terms of RMSD, DNM1 ^p.Glu468*^ after initial increase and decrease showed stable behavior till 100 ns MD run while DNM1^WT^ revealed stable behavior during the entire 100 ns MD simulation except for some minor deviations at 55–75 ns of MD run. The RMSF trajectory for DNM1^p. Glu468*^ showed fluctuations at the residues 105–107, 110–113, and 325 while the residues 114–324 showed stability along with native DNM1 that showed major and minor fluctuations at residues 648–652 and 740–750, respectively. Rg denotes the overall spread of the molecule, the average values of Rg were indicated in the range of 29.5–31.0 Å and 31.0–32.0 Å for DNM1 ^p. Glu468*^ and wild type, respectively. It is clear from the Rg calculation that the wild-type system revealed more compactness as compared to the mutant form during the MD simulation. Overall, an MD run demonstrated that the non-sense variant in DNM1 protein leads to instability of the p. Glu468* system in comparison to the wild-type protein as manifested by varied trajectories of RMSD, RMSF, and Rg, respectively. In addition, functional validation of this non-sense variant in the *DNM1* gene using RT-qPCR approach revealed a decreased expression of the gene in the index patient signifying loss-of-function effect (45). For families impacted by rare inheritable diseases, proper genetic counseling is crucial. Additionally, prenatal genetic screening/diagnosis is the most effective management method for these medical conditions, for which there is no cure at this time (46).

In summary, we reported a novel non-sense variant in the *DNM1* leading to severe neurodevelopmental phenotype in the patient that includes early-onset epilepsy, hypotonia, intellectual disability, seizures episodes, and pronounced developmental delay. This study will aid in genetic counseling and bringing awareness regarding the disease in the common population. The study also adds to the variant spectrum of *DNM1* and contributes to the evaluation of genotype–phenotype correlation.

## Data Availability

The datasets presented in this study can be found in online repositories. The names of the repository/repositories and accession number(s) can be found in the article.
